# Efficacy analysis of quadruple nerve decompression surgery for lower limb diabetic peripheral neuropathy

**DOI:** 10.3389/fsurg.2025.1702779

**Published:** 2026-02-05

**Authors:** Yong Zhang, Zonghan Li, Tianyi Ma, Xiaodong Xu, Jicheng Li, Rufei Dai, Jiawei Shen

**Affiliations:** 1 Department of Neurosurgery, The Second Affiliated Hospital of Xuzhou Medical University, Xuzhou, Jiangsu, China; 2 Graduate School, Xuzhou Medical University, Xuzhou, Jiangsu, China

**Keywords:** quadruple nerve decompression, diabetic peripheral neuropathy, painful peripheral neuropathy, nerve compression, SCV, TCSs, VAS

## Abstract

**Objective:**

To explore the efficacy of quadruple nerve decompression in treating painful diabetic peripheral neuropathy (PDPN) of lower extremity, and to evaluate its clinical value in pain relief and sensory recovery.

**Method:**

A retrospective analysis was performed on 26 PDPN patients (45 sides), all of whom underwent quadruple nerve decompression, including release of the common peroneal nerve (CPN), superficial peroneal nerve (SPN), deep peroneal nerve (DPN), and tibial nerve (TN). Changes in the Visual Analog Scale (VAS) score, two-point discrimination (TPD), sensory nerve conduction velocity (SCV), and Toronto Clinical Scoring System (TCSS) score were evaluated by comparing preoperative values with those at an average of 30.46 months postoperatively. Statistical analysis was conducted using the paired *t*-test.

**Results:**

Postoperative VAS scores were significantly reduced, from 7.31 ± 1.62 to 2.51 ± 1.47 (*P* < 0.001), with 93.3% of limbs achieving at least 50% pain relief. TPD showed significant improvement, decreasing from 13.80 ± 3.01 mm to 7.49 ± 2.07 mm (*P* < 0.001), and 68.9% of patients returned to normal levels. The proportion of nerves showing an SCV improvement of ≥5 m/s ranged from 64.4% to 75.6%. TCSS scores shifted from all being grade III before surgery to mild or moderate in 93.3% of cases. No severe complications were observed postoperatively.

**Conclusion:**

Significant pain relief and improvement in sensation and nerve function have been achieved in patients with PDPN through quadruple nerve decompression, which addresses multiple potential nerve entrapment sites. This procedure, building upon existing evidence, demonstrates sustained efficacy in pain relief and sensory recovery over a median 30-month follow-up, offering a refined surgical option for patients with refractory PDPN who have failed conservative management.

## 1 Introduction

PDPN of the lower extremities is characterized by persistent distal burning pain and sensory loss, with an estimated prevalence of 16%–26% among people with diabetes. Conventional pharmacological analgesia provides ≥50% pain relief in only 30%–40% of patients and fails to halt the progressive decline in nerve function (1). Histological studies have confirmed that chronic hyperglycemia leads to collagen thickening of the perineurium and epivascular membrane, contributing to anatomical stenosis at multiple sites, including the tibial and fibular tunnels (2). This results in nerve ischemia and subsequent axonal degeneration, which are critical drivers of symptom progression (2). Professor Dellon was the first to introduce triple nerve decompression for diabetic peripheral neuropathy. Although the traditional Dellon triple procedure is relatively less invasive, it may overlook the fibular tunnel or other entrapment sites. Quadruple nerve decompression enhances the procedure by adding decompression of the SPN, aiming to restore nerve blood flow, alleviate neuropathic symptoms, and relieve pain by simultaneously releasing the narrowed channels of the CPN, SPN, DPN, and TN (3). Currently, debate continues over the impact of increased incision number and extent on postoperative recovery, and few studies have evaluated the quadruple procedure for PDPN. This study aims to evaluate the additional benefits of quadruple nerve decompression in early postoperative pain relief and sensory recovery in patients with PDPN, using the VAS, TPD, and TCSS to objectively assess efficacy and provide evidence for clinical pathway selection (4). While preliminary RCTs have explored quadruple decompression, this study aims to extend the evidence base by evaluating its long-term multidimensional outcomes and real-world applicability in a strictly selected cohort.

## 2 Materials and methods

### 2.1 Case selection

This retrospective study included 26 patients diagnosed with diabetic peripheral neuropathy, all of whom met the diagnostic criteria outlined in the 2025 American Diabetes Association (ADA) Standards of Medical Care in Diabetes (5). The diagnosis of PDPN was made in accordance with the guideline standards established by the International Association for the Study of Pain (6–8). Among the 26 patients, there were 13 males and 13 females, aged between 39 and 78 years, with a mean age of 61.54 ± 10.24 years. The median duration of diabetes was 10.5 years (interquartile range [Q1, Q3]: 7.75–15.0), and the median duration of painful diabetic neuropathy was 8.0 years (Q1, Q3: 5.0–12.5). Ethical approval for this study was granted by the Second Affiliated Hospital of Xuzhou Medical University Ethics Committee (approval number: 2025080201).

Inclusion criteria:

① Underwent quadruple decompression of the CPN, TN, SPN, and DPN; ② A preoperative TCSS score of ≥6 and a positive result on the Michigan Neuropathy Screening Instrument (9); ③ Abnormal nerve conduction in the lower extremity confirmed by nerve electrophysiological examination, presenting as numbness, pain, or stocking-glove sensory impairment, with no improvement after conservative treatment (e.g., medication); ④ Preoperative hemoglobin A1c ≤ 69 mmol/mol (8.5%) and fasting blood glucose maintained at 6.0–10.0 mmol/L to ensure a metabolic state appropriate for surgery (10); ⑤ Evidence of nerve entrapment on imaging (e.g., musculoskeletal ultrasound) with a positive Tinel's sign; ⑥ No history of local anesthetic allergy and absence of active infection or ulcer in the surgical area; ⑦ Signed surgical and clinical research informed consent.

Exclusion criteria: ① A previous history of lower-extremity nerve transection injury or surgery, with muscle atrophy observed in the area innervated by the target nerve; ② Acute or progressive systemic conditions, including ACS, decompensated heart failure, progressive hepatorenal failure, and active infection; ③ Coagulation abnormalities were defined as an INR greater than 1.5 or a platelet count below 100 × 10^9^/L; patients requiring uninterrupted long-term anticoagulation or dual antiplatelet therapy were excluded; ④ Poor metabolic control is indicated by preoperative hemoglobin A1c levels above 69 mmol/mol (8.5%) or fasting blood glucose levels below 6.0 mmol/L or above 10.0 mmol/L, which may increase the risk of intraoperative hypoglycemia, postoperative infection, delayed wound healing, and progression of neuropathy; ⑤ pregnancy, lactation, or planned pregnancy; ⑥ inability to tolerate surgery or to complete follow-up.

### 2.2 Neuroelectrophysiological examination data

Nerve electrophysiological examinations of 45 affected limbs all indicated impairment of the CPN, SPN, DPN and TN function; their SCV was either below the normal lower limit or at a borderline low value, with or without prolonged H-reflex and F-wave latency and abnormal amplitude. When SCV results were indeterminate, immediate retesting was performed with strict control of skin temperature ≥32 °C, standardized distances and superimposed averaging; if still indeterminate, additional tests such as inching study, magnetic resonance neurography or skin-biopsy small-fiber quantification were conducted to differentiate between entrapment and metabolic damage (11).

### 2.3 Surgical technique

**Figure 1:**
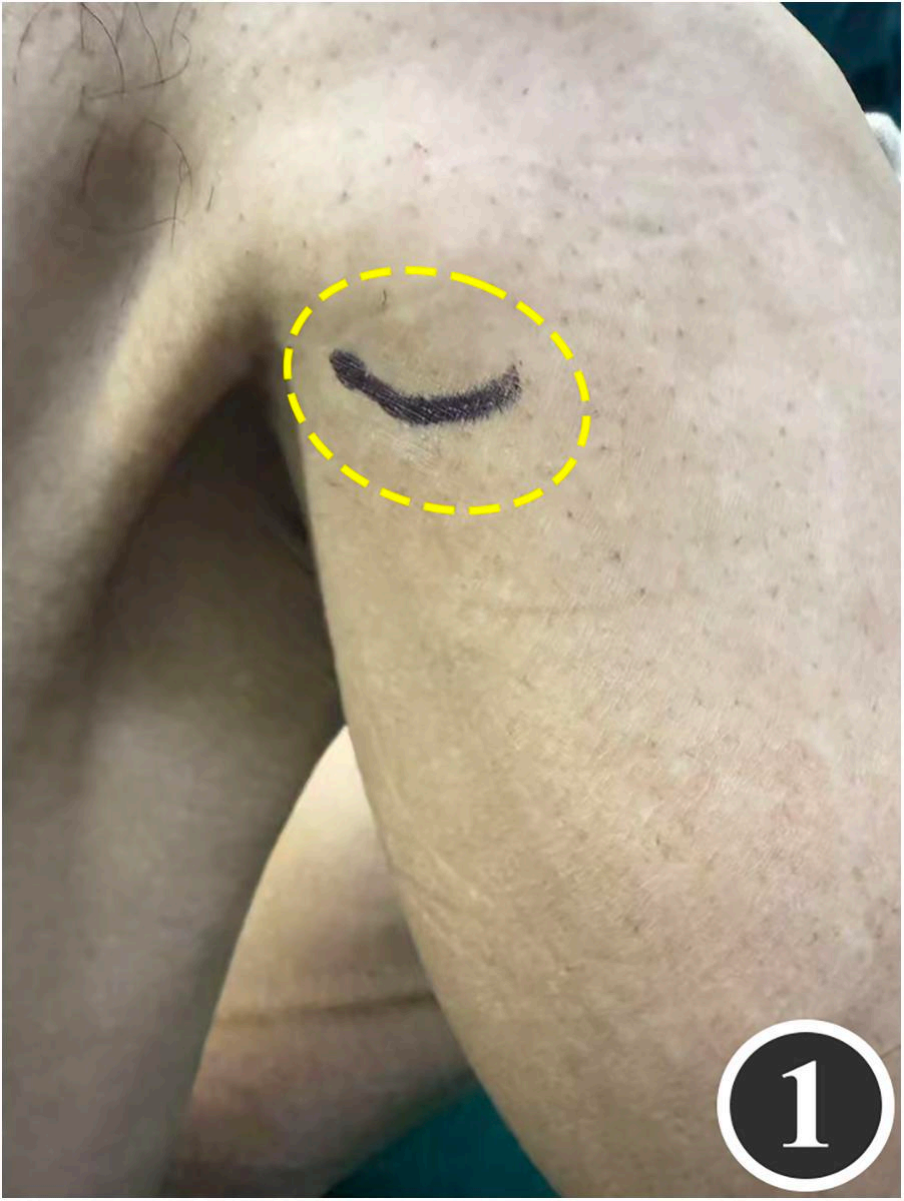
Marking of the decompression incision for the CPN.

**Figure 2:**
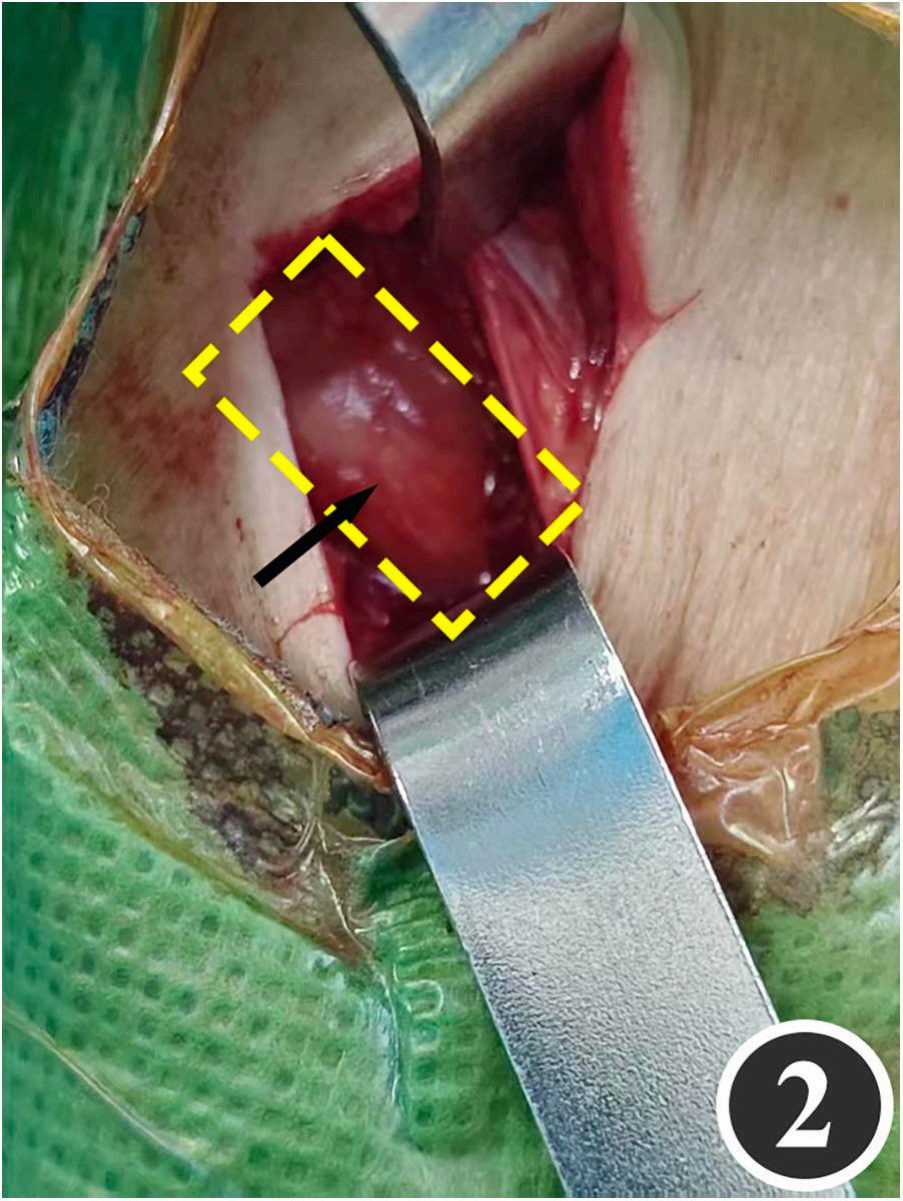
The CPN after release is indicated by the arrow.

**Figure 3:**
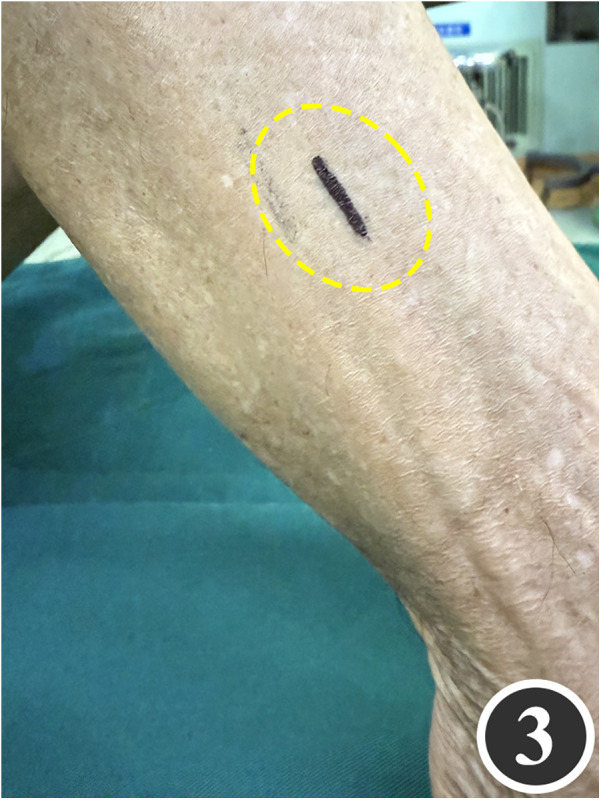
Marking of the decompression incision for the SPN.

**Figure 4:**
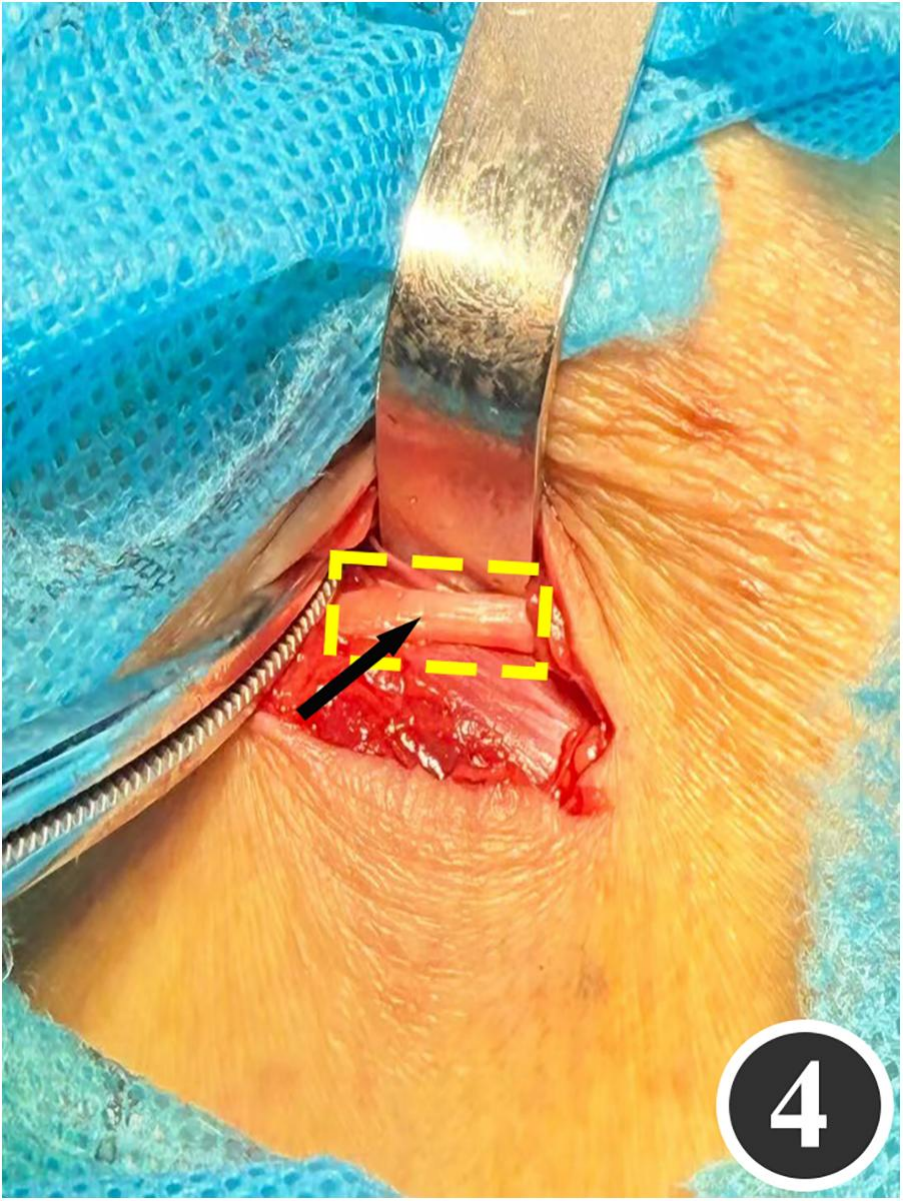
The SPN, as indicated by the arrow, has been released.

**Figure 5:**
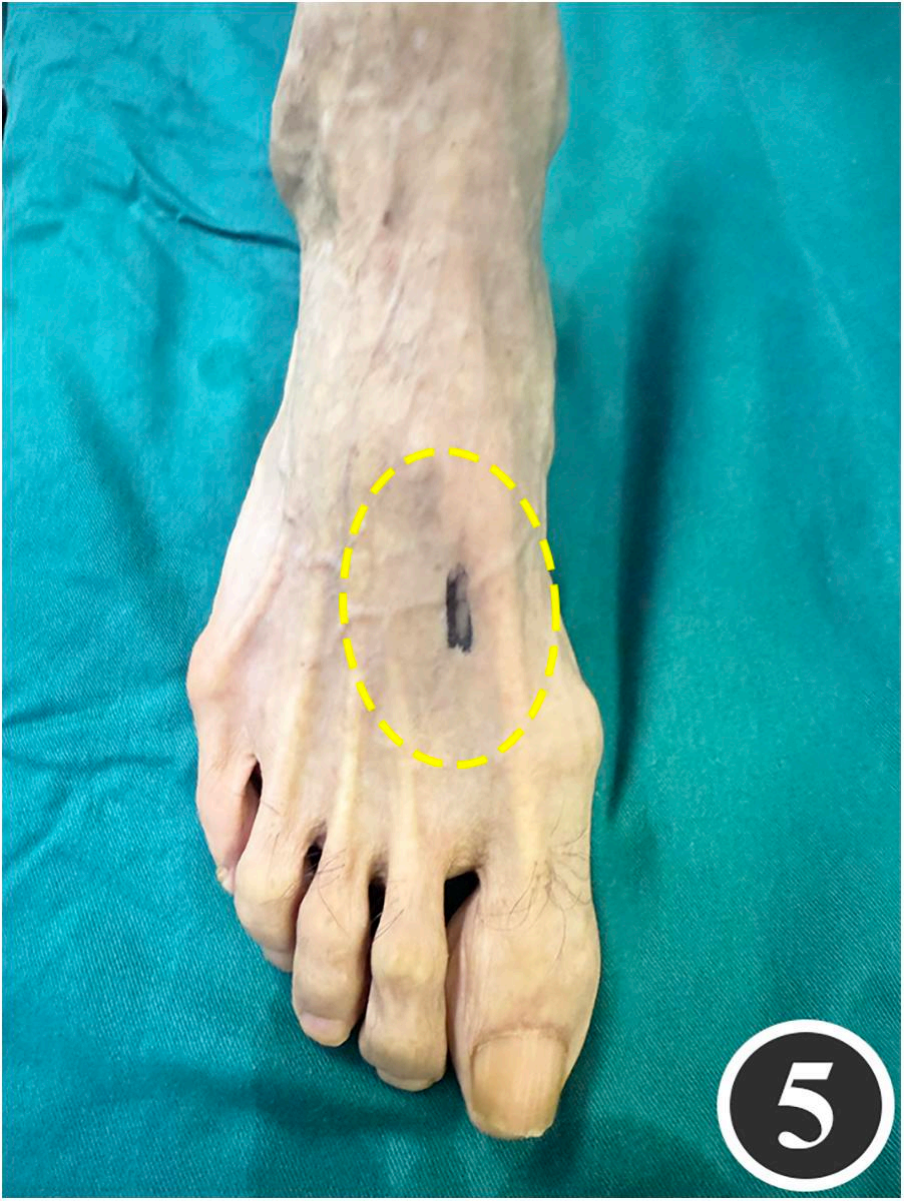
Marking of the decompression incision for the DPN.

**Figure 6:**
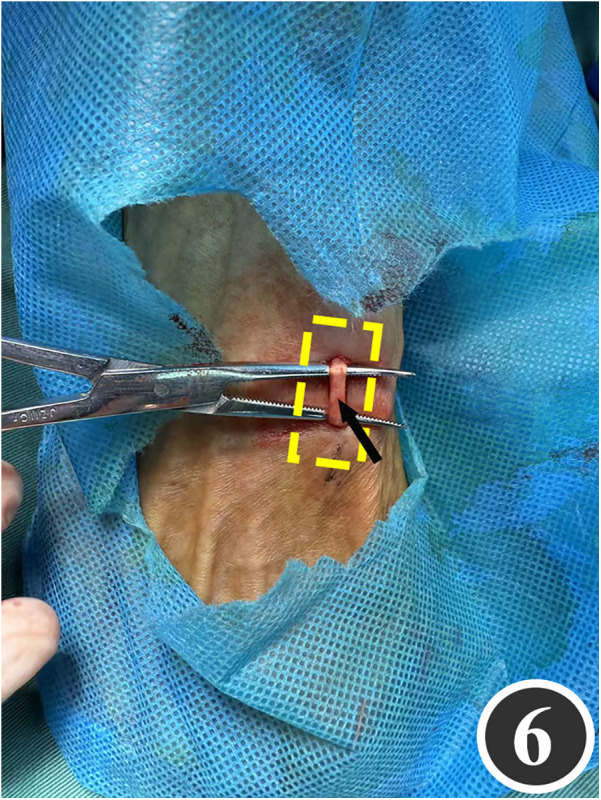
The arrow points to the released extensor pollicis brevis tendon and surrounding tissues, through which the DPN has been fully decompressed.

**Figure 7:**
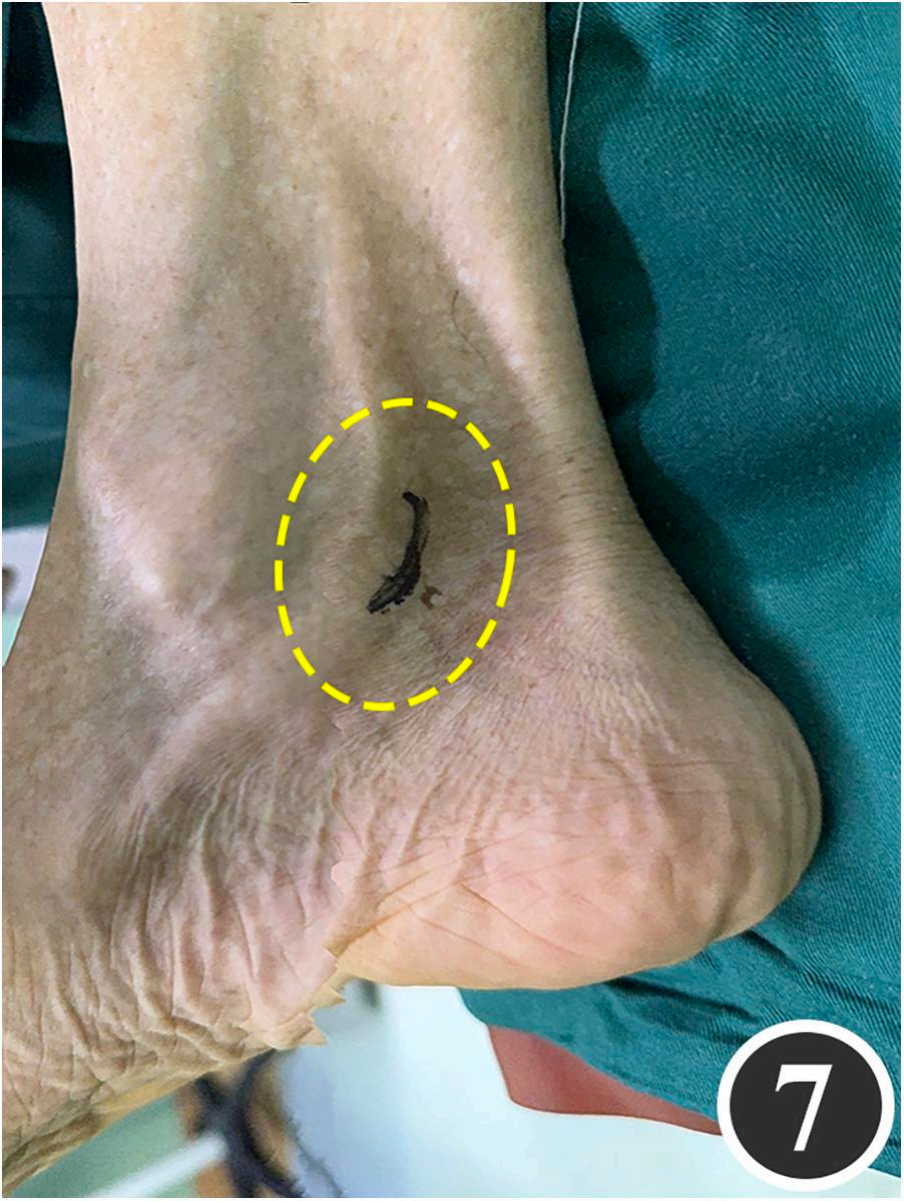
Marking of the TN decompression incision.

**Figure 8:**
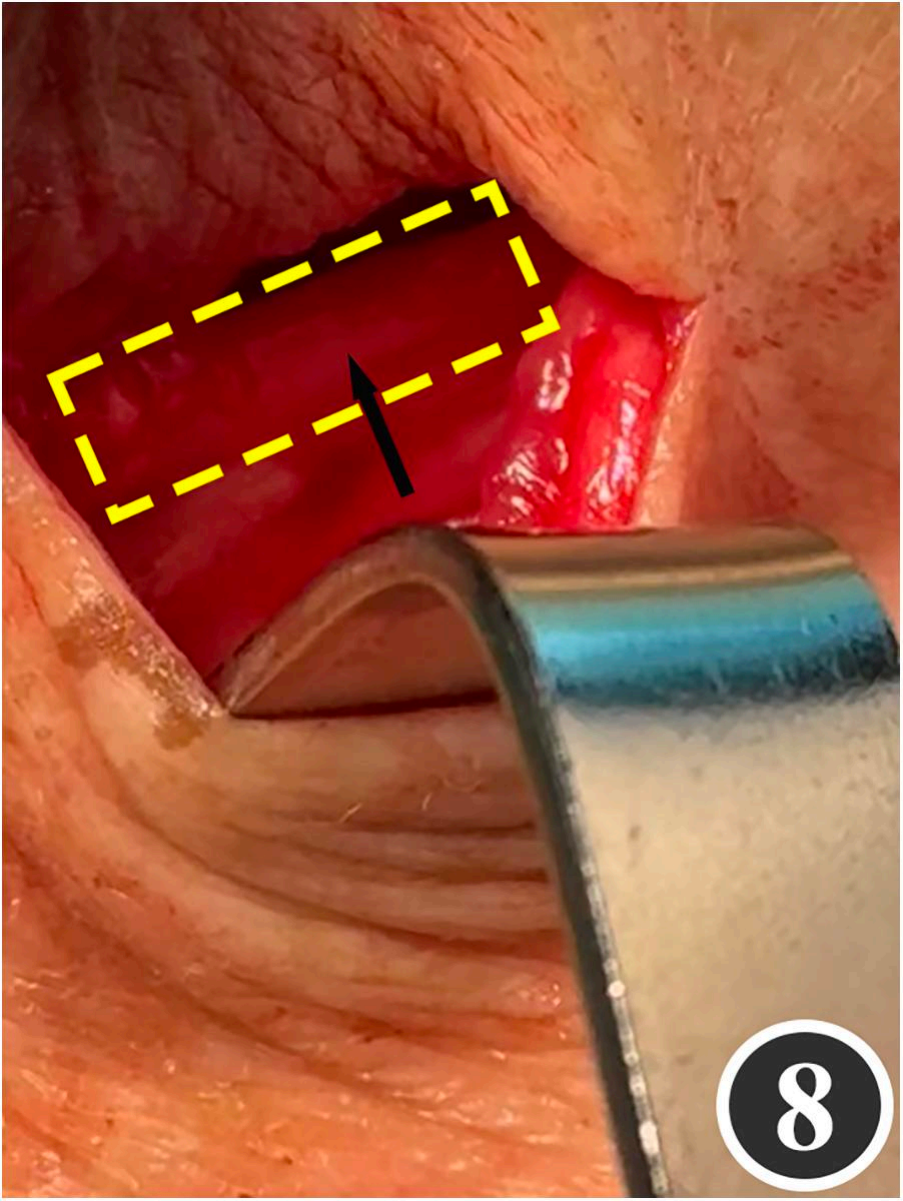
The arrow shows the direction of the decompressed TN.

All patients underwent quadruple nerve decompression of the lower extremity, including unilateral or bilateral CPN, SPN, DPN and TN trunks and their branches. The surgery was performed by an experienced neurosurgeon. Prior to the procedure, thorough communication was conducted with the patient, and the side with earlier or more severe symptoms was treated first; after about 4 weeks postoperatively, once the function of the affected limb had recovered and daily activities had returned to normal, treatment of the contralateral side was scheduled. All surgeries were performed under endotracheal intubation or laryngeal mask anesthesia combined with intravenous and inhalation anesthesia, with a lower-extremity tourniquet routinely applied at a pressure of 250–260 mmHg (1 mmHg = 0.133 kPa). The steps for each nerve decompression were as follows: ① CPN: the patient was positioned supine, with the hip and knee on the operative side flexed at 90° so that the lower leg was perpendicular to the bed. A 3–4 cm curved incision was made posteroinferior to the fibular head, which served as the landmark (Figure 1). The CPN was first identified and dissected posterior-inferior to the fibular head, then carefully separated anteriorly. The peroneus longus tendon and surrounding fibrous bundles were exposed and released (Figure 2), with partial removal of tendinous tissue performed if necessary. Dissection was extended proximally to the biceps femoris aponeurosis, followed by internal neurolysis under the microscope. ② SPN: an incision was made parallel to the fibula at the anterolateral aspect of the lower leg, one-third distal and 10–12 cm above the lateral malleolus (Figure 3); the superficial fascia was incised, and the entrapment fascia outlet was identified and severed; the deep fascia was freed and opened 10 cm on both sides, and the SPN was fully released (Figure 4). ③ DPN: a longitudinal 4 cm incision was made on the dorsum of the foot between the first and second toes (Figure 5). The superficial fascia was freed, traction applied to protect the medial cutaneous nerve of the dorsum of the foot, the deep fascia incised, the extensor tendon of the extensor hallucis brevis exposed, both the proximal and distal sides of the tendon fully freed, and the DPN and its surrounding tissues thoroughly released (Figure 6). ④ TN: an arcing incision was made posterior to the medial malleolus along the course of the TN (Figure 7); part of the flexor retinaculum at the medial malleolus was resected to expose the posterior tibial neurovascular bundle; the superficial fascia of the abductor hallucis muscle was distally separated to expose its main trunk, along with the medial and lateral plantar nerves and the heel nerve (Figure 8); full relaxed decompression was performed. After the tourniquet was released, complete haemostasis was achieved. The incision was sutured intermittently in layers, and a cotton pad was moderately compressed and bandaged.

### 2.4 Efficacy evaluation indicators

① Pain assessment: The VAS was used to assess pain intensity in patients with painful diabetic peripheral neuropathy; the score ranges from 0 to 10 (0 = no pain, 10 = severe pain) (8). ② Somatosensory function: The minimum distance at which the two tips of the TPD device could be perceived on the plantar skin of the great toe was recorded. A TPD ≤8 mm was considered normal, 8–14 mm mild impairment, 14–20 mm significant impairment, and >20 mm complete loss of sensation (12). ③ SCV: Electrophysiological testing of lower-extremity SCV was performed for the CPN, TN, SPN, and DPN. ④ Severity of neuropathy: The TCSS was used to comprehensively evaluate neurological symptoms, reflexes, and sensory function. Scores of 0–5 indicate no neuropathy, 6–8 mild, 9–11 moderate, and 12–19 severe (13).

### 2.5 Follow-up methods

Follow-up examinations were performed at 1, 3, 6, and 12 months, and then annually thereafter. Surgical outcomes were comprehensively assessed each time using the VAS pain score, TCSS neuropathy score, SCV, and TPD. At each follow-up, it was recorded whether the VAS was ≤3 points and the date on which this threshold was first achieved, in order to calculate the duration of pain relief in months.

### 2.6 Statistical methods

Data were analysed with SPSS version 27.0. The Shapiro–Wilk test was used to assess normality; *P* > 0.05 indicated a normal distribution, and results are presented as mean ± standard deviation (x̅ ± s). Paired data were compared with the paired *t*-test. Multiple comparisons of SCV among the four nerves were adjusted by Bonferroni correction (*α*′ = 0.0125). Continuous variables are expressed as mean ± standard deviation (x̅ ± s), and categorical variables as counts and percentages. A *P*-value < 0.05 was considered statistically significant.

## 3 Result

### 3.1 Wound healing status

All 26 surgeries were completed successfully. Intraoperative confirmation showed nerve compression and fixation at the fibular tunnel, dorsum of the foot, and tarsal tunnel, with adhesions present at the SPN penetration site. Postoperatively, only three cases experienced delayed healing of the posterior incision at the medial malleolus, and no other complications were observed.

### 3.2 Efficacy evaluation

**Figure 9:**
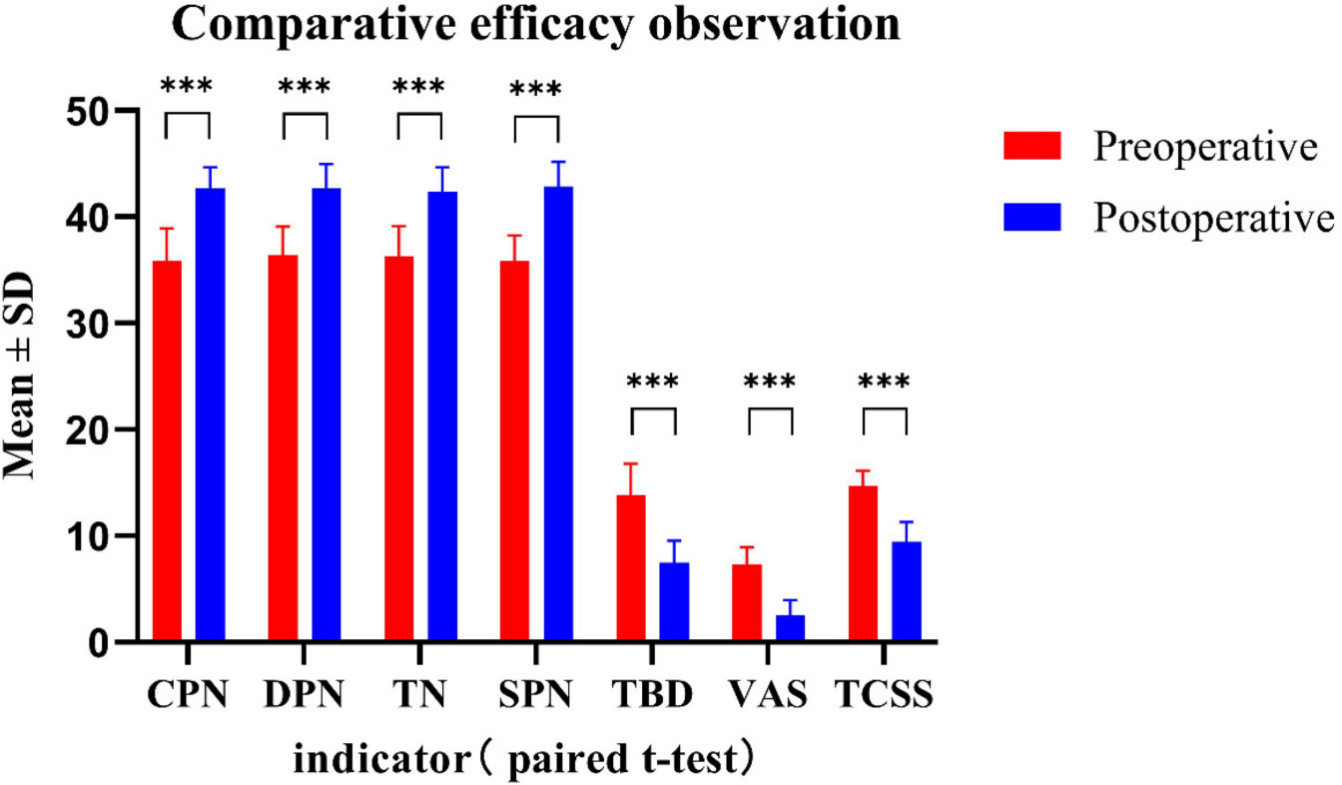
Efficacy was observed and compared before and after quadruple nerve decompression surgery in 26 patients (45 limbs) with PDPN of the lower extremities ($\bar{x}$ ± s). The VAS is used to measure pain intensity, TPD assesses sensory function, and TCSS evaluates clinical symptoms (*P < 0.05; **P < 0.01; ***P < 0.001).

**Figure 10:**
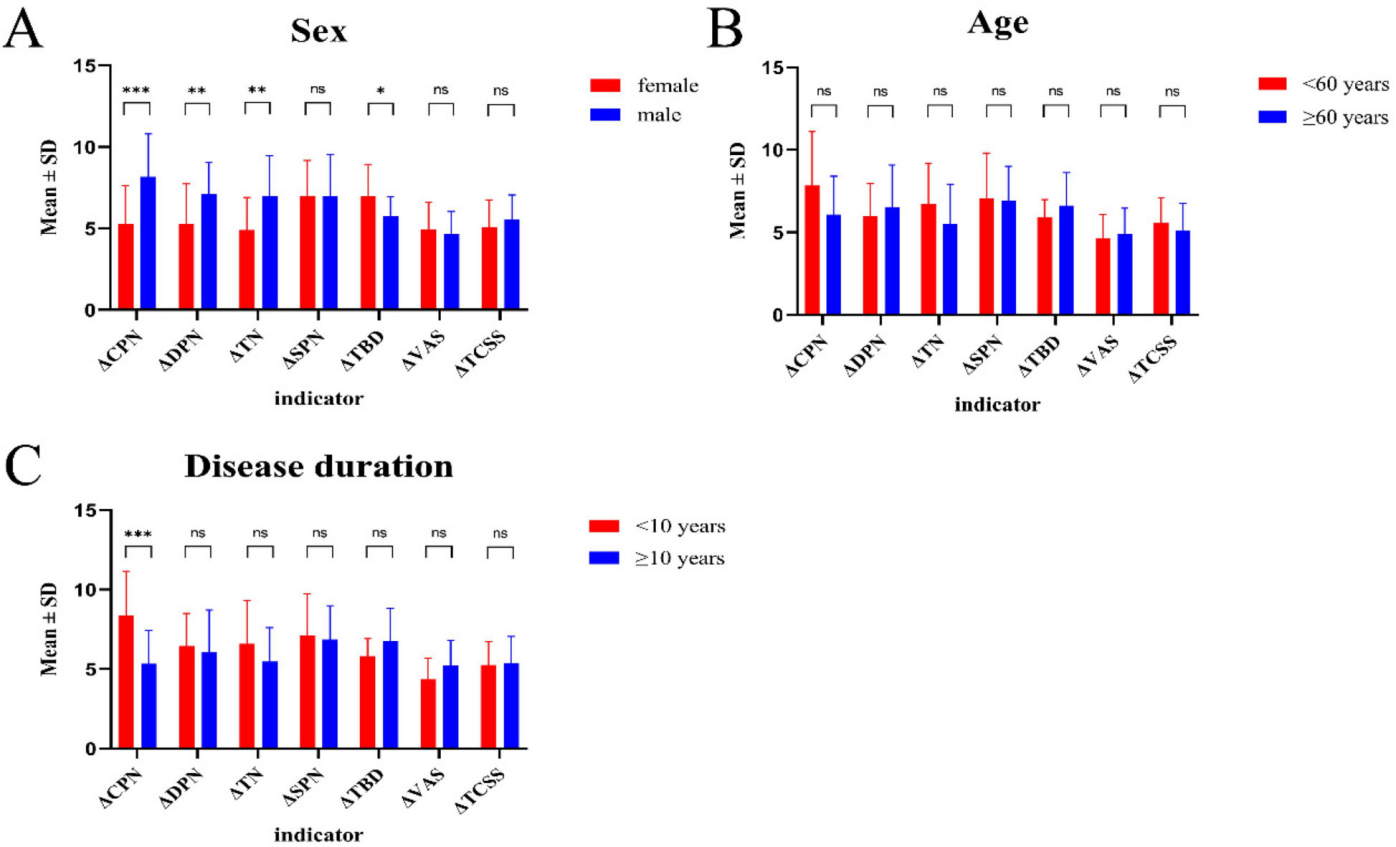
**(A)** Gender-based, **(B)** age-based, and **(C)** diabetes-duration-based subgroup analyses of ΔCPN, ΔDPN, ΔTN, ΔSPN, ΔTBD, ΔVAS and ΔTCSS in 26 patients (45 limbs) with lower-extremity PDPN. Differences between pre- and post-operative values are shown (*P < 0.05; **P < 0.01; ***P < 0.001).

The average follow-up period for the 26 patients was 30.46 ± 8.90 months (range 15–48 months). At the last follow-up, a significant reduction in VAS scores was observed in 45 lower extremities (*P* < 0.001); 44.4% (20/45) showed an improvement of ≥70% and 93.3% (42/45) an improvement of ≥50%. Pressure-sensation TPD measurements in 45 lower-extremity sides demonstrated a significant overall postoperative improvement (*P* < 0.001); 31 sides returned to normal and 35.6% (16/45) showed an improvement of ≥50%. Postoperative increases in SCV of ≥5 m/s were recorded in 75.6%, 71.1%, 68.9% and 64.4% of cases for the CPN, SPN, DPN and TN, respectively; all differences were statistically significant (*P* < 0.001). TCSS scores improved significantly: all pre-operative grade-III cases were re-classified post-operatively as 9 mild, 14 moderate and 3 severe (grade III) (*P* < 0.001; Figure 9). Males exhibited significantly greater post-operative improvement than females, particularly for ΔCPN, ΔDPN and ΔTN (*P* < 0.01); no sex-related differences were detected in TCSS score changes (*P* > 0.05; Figure 10). The efficacy of quadruple-nerve decompression was significant (Table 1).

**Table 1:** All primary outcome measures showed a Cohen's d greater than 2.0, indicating an extremely large effect size. This suggests that quadruple nerve decompression produces a highly significant clinical improvement in relieving pain, enhancing sensory function, and nerve conduction.

Biomarker	Preoperative mean ± SD	Postoperative mean ± SD	ΔPre-Post	Cohen's d	95% CI
VAS score	7.31 ± 1.62	2.51 ± 1.47	4.80	3.20	2.45–3.94
TPD (mm)	13.80 ± 3.01	7.49 ± 2.07	6.31	3.70	2.86–4.55
SCV (m/s)					
CPN	35.91 ± 3.03	42.73 ± 1.96	6.82	2.38	1.79–2.96
SPN	35.87 ± 2.41	42.84 ± 2.36	6.97	2.96	2.26–3.65
DPN	36.42 ± 2.70	42.67 ± 2.29	6.25	2.67	2.03–3.31
TN	36.33 ± 2.80	42.36 ± 2.32	6.03	2.43	1.83–3.02
TCSS score	14.73 ± 1.43	9.42 ± 1.90	5.31	2.57	1.95–3.19

### 3.3 Duration of pain relief

Among the 45 affected limbs, the pre-operative VAS score was 7.31 ± 1.62. All limbs exhibited burning pain on the dorsum of the foot extending to the plantar region, which worsened at night and upon weight-bearing; no concomitant radicular or central pain was observed. At the last follow-up (mean 30.46 ± 8.90 months), 88.9% (40/45) maintained a VAS score ≤3, confirming medium- to long-term stability of pain relief. Further analysis revealed that only two cases (4.4%) experienced pain rebound at 18 months post-operatively (VAS increased from 2 to 5); both were associated with elevated blood glucose and weight gain, suggesting that poor metabolic control may be a key factor in pain recurrence.

### 3.4 Postoperative neural edema monitoring

High-resolution nerve ultrasound was repeated in 26 patients 12 months post-operatively to assess whether the previously decompressed nerves—particularly the SPN—showed recurrent enlargement of cross-sectional area (CSA) or increased epineurial echogenicity. None of the CSA values returned to pre-operative levels; mild re-stenosis was observed in only one SPN, without clinical recurrence. No significant epineurial thickening or new adhesions were detected, indicating that structural re-oedema did not occur after decompression.

## 4 Discussion

### 4.1 Challenges in treating diabetic peripheral neuropathy and the need for surgical intervention

Infection, ulceration and even limb amputation can result from diabetes-related lower-extremity peripheral neuropathy, making it one of the most serious complications encountered in diabetic patients (14, 15). Distal symmetrical polyneuropathy is the primary manifestation, characterised by symmetrical pain and numbness in the distal limbs together with impaired pain and temperature sensation. In severe cases muscle weakness, atrophy or deformity may develop, accompanied by glove-and-stocking sensory loss and restricted movement (16, 17). Current treatments consist mainly of antidepressants, potent analgesics and topical agents such as capsaicin ointment; more recently, monochromatic near-infrared irradiation has been trialled to relieve symptoms, but results remain limited (15, 18).

### 4.2 The “double compression” mechanism: the theoretical foundation of nerve decompression surgery

Since 1992, the use of multiple-nerve decompression to relieve pain and restore sensation has been documented in the surgical literature. Peripheral nerves in patients with diabetes are susceptible to a “double-compression” mechanism involving both internal nerve oedema and external compression by anatomical channels. This combined effect impairs the nerve's ability to self-repair and reduces its blood supply, ultimately producing the symptoms of neuropathy (19, 20). The precise pathways remain incompletely defined. Previous studies have shown that onset and progression are associated with metabolic dysfunction, microcirculatory impairment, immune responses and microbial factors. Hyperglycaemia-induced metabolic disturbance and microangiopathy cause intraneural oedema, increasing nerve cross-sectional area (21). Advanced glycation end-product (AGE) cross-linking thickens and stiffens tendon sheaths, ligaments and fibro-osseous tunnels (tarsal, fibular, carpal), decreasing luminal volume. The hypertrophic ligament exerts a “clamping” effect on the swollen nerve, obstructing intraneural microcirculation and producing “secondary ischaemia” (22). Focal demyelination and axonal degeneration subsequently develop in nerves already injured by chronic hyperglycaemia under this dual compressive insult (23).

### 4.3 Limitations of the dellon triple procedure and theoretical advances of the quadruple procedure

A comprehensive theory on the application of peripheral nerve decompression surgery for treating diabetic peripheral neuropathy was first developed by Professor Dellon from the United States. This theory was validated using animal models, addressing a gap in the field and establishing the theoretical foundation for the global adoption of peripheral nerve decompression surgery. An increasing number of researchers have subsequently applied this procedure in patients, and its effectiveness in alleviating the symptoms of diabetic peripheral neuropathy has been demonstrated. However, the number of cases performed remains limited, and the available evidence-based medicine evidence is insufficient. As a result, some scholars argue that the therapeutic efficacy of this procedure has yet to be conclusively validated. Currently, the debate surrounding this procedure continues to intensify (19). The Rozen 2024 DNND trial found no significant difference in pain relief between sham surgery and actual decompression, indicating that at least half of the observed high efficacy of diabetic nerve decompression surgery may be attributed to the placebo effect (24). However, increasing evidence suggests that this improvement is more likely attributable to the mechanism of central nervous system collateral regeneration and the release of systemic neurotrophic factors. Therefore, the negative conclusion drawn by Rozen et al. based on the “sham surgery control” does not truly refute the clinical efficacy of nerve decompression surgery.

### 4.4 Evidence gaps and the clinical significance of quadruple therapy exploration

Typical symptoms are observed in patients with lower-extremity diabetic peripheral neuropathy, including sensory disturbances in the regions innervated by the CPN and TN. “Sock-and-glove” type paraesthesia is commonly experienced, which requires combined decompression of multiple nerves at various sites in the lower extremity. Lower-extremity triple decompression is a combined procedure involving three anatomical sites: the medial malleolus TN, fibular tunnel CPN, and dorsal pedal DPN along with its branches. First pioneered by US Professor Dellon, this technique is known as the “Dellon triple procedure”. It has been widely adopted nationally and internationally and remains a standard operation for peripheral nerve decompression in patients with diabetic lower-extremity peripheral neuropathy (25). Dissatisfaction with sensory recovery in the dorsum of the foot and calf has been reported by most patients after Dellon triple release; this may be attributed to the procedure's failure to address potential additional sites of nerve compression. It has been confirmed that in patients with diabetic peripheral neuropathy of the lower extremity, compression is not confined to the fibular tunnel at the lateral knee affecting the CPN, dorsal pedal DPN, medial malleolus TN and its three branches, but also involves the lower anterior one-third of the leg SPN (19).

### 4.5 The mechanism of SPN entrapment and the theoretical foundation of quadruple nerve decompression

The SPN, a terminal branch of the CPN, originates posterior to the fibular head. It initially traverses the deep surface of the peroneus longus, then descends between the peroneus longus and brevis. Approximately 10–15 cm above the ankle it pierces the deep fascia to become subcutaneous and divides into the medial dorsal cutaneous nerve and the intermediate dorsal cutaneous nerve. The sensory territory of the SPN covers the lower two-thirds of the anterolateral leg and most of the dorsum of the foot, except the first web space and the lateral border of the fifth toe (26, 27). Under physiological conditions, compression along its course is rare. In diabetic peripheral neuropathy, however, nerve oedema, effusion and adhesions occur together with fascial and retinacular thickening, producing compression and traction at the nerve's exit and causing sensory disturbance in its distribution. These findings indicate that the SPN in the lower anterior third of the leg is also a vulnerable entrapment site. It is therefore theoretically feasible to include four sites in lower-extremity peripheral nerve decompression: the CPN at the fibular tunnel lateral to the knee, the TN at the medial malleolus, the dorsal pedal DPN and the SPN in the lower anterior third of the leg. This procedure was first developed and promoted by the team led by Professor Zhang Li at China–Japan Friendship Hospital; it provides more comprehensive decompression of potential entrapment sites and thereby broader symptom relief (28).

### 4.6 Multimarker validation of quadruple therapy benefit

The quadruple decompression procedure represents an innovative modification of the Dellon triple decompression. Although triple-nerve decompression is widely practised, randomised controlled trials (RCTs) with traceable data remain rare. van Maurik et al. (29) reported that, in a study of 42 patients with unilateral PDPN, significant pain relief on the surgical side was observed in 73.7% of cases one year post-operatively using a self-controlled design (surgical vs. contralateral side); however, neither SCV nor TCSS was evaluated, limiting assessment of the degree of nerve-function recovery. In a single-blind, parallel-group RCT conducted by Best et al. (19), 22 patients were randomly assigned to surgery (*n* = 12) or observation (*n* = 10); significant improvements in VAS and the NeuroQoL pain subscale were observed in the surgery group, but SCV and TCSS were not systematically assessed and the sample size was small. A multicentre RCT protocol comparing triple therapy with medication was published by Liao et al. (30), aiming to enrol 74 patients with PDPN; the primary end-point is a 50% reduction in VAS, while secondary outcomes including TCSS, SCV and TPD will be systematically assessed, but results have not yet been released.

### 4.7 Comparison with previous RCTs: the gap addressed by this study

Although quadruple nerve decompression has been preliminarily applied in some studies (e.g., the randomized controlled trial by van Maurik et al.) (31), several critical gaps remain in the current body of evidence: ① a lack of long-term efficacy data, with most studies reporting a follow-up duration of ≤12 months; ② insufficient integration of multi-dimensional outcome indicators, such as the failure to simultaneously assess four core metrics—VAS, TPD, SCV and TCSS; ③ limited exploration of the relationships among gender differences, metabolic control and treatment efficacy. This study, through a medium- to long-term follow-up averaging 30.5 months, systematically validated the sustained efficacy of quadruple decompression in pain relief, sensory recovery and improvement of nerve conduction function. It also, for the first time, reported a gender difference in postoperative outcomes, with male patients showing significantly greater improvement in SCV compared with female patients. In addition, we introduced postoperative ultrasound monitoring of nerve re-edema as a means to assess structural stability, providing morphological evidence for the durability of surgical decompression. These findings not only reinforce the clinical rationality of quadruple decompression but also offer practical guidance for individualized patient selection and postoperative management.

### 4.8 Efficacy stability and risk of recurrence

A total of 26 patients with lower-extremity PDPN were strictly selected for this study, all of whom met the surgical indications. In addition to the typical entrapment signs of the fibular-tunnel CPN, dorsal-pedal DPN and TN at the medial malleolus, significant pathological changes—including fascial thickening, nerve oedema and adhesions—were observed at the SPN exit in the anterior lower third of the calf. Inclusion of the SPN in the decompression field produced marked post-operative improvement in sensation over the dorsum of the foot and the anterolateral leg. Compared with the “subjective sensory improvement of 67%” reported by Dellon in 1992 (23), the present study quantified the effect: VAS documented significant pain relief in 93.3% of limbs, far exceeding Dellon's original figure; TPD showed restoration of normal sensation in 68.9%, providing objective confirmation; SCV improvement rates for the four nerves ranged from 64.4% to 75.6%, supplying electrophysiological evidence; and 93.3% of limbs showed post-operative down-grade of TCSS from grade III to mild or moderate, indicating global neuropathy alleviation. At 30 months' follow-up, 88.9% of limbs maintained VAS ≤ 3, demonstrating stable mid- to long-term results. Subgroup analysis revealed better functional recovery in men than in women, whereas age and pre-operative TCSS score had no significant influence on efficacy. Post-operative pain relief was not always permanent; the small risk of recurrence appeared related to poor metabolic control. No re-development of nerve oedema was detected during surveillance, and imaging follow-up confirmed sustained decompression, further supporting the capacity of surgery to relieve oedema-mediated symptoms arising from the “double-crush” mechanism.

### 4.9 Surgical safety and incision healing

No incisional infections were observed post-operatively; however, delayed healing of the medial malleolus incision occurred in three patients, probably because of poor local perfusion and continuous motion across the joint. No other incisions showed delayed healing. Previous work has shown that larger wounds heal more slowly in diabetic patients receiving standard care (32), yet whether a greater number of incisions increases the risk of delayed healing or infection remains uncertain. Additional cases are required to provide stronger evidence.

### 4.10 Limitations of the study and future directions

The quadruple procedure, which builds upon Dellon's triple procedure by incorporating SPN decompression, is designed to address a broader range of potential entrapment points and may enhance efficacy to a certain extent. It appears particularly beneficial for patients presenting with extensive symptoms and multiple-nerve involvement. However, as this study was a retrospective analysis without a control group—such as a sham-surgery or conservative-treatment group—the influence of the placebo effect or other non-surgical factors on efficacy could not be ruled out. In addition, the limited number of cases may restrict the generalisability and statistical power of the findings. To more robustly assess its efficacy and safety, further investigation through large-sample, multicentre, randomised controlled trials is warranted to provide higher-level evidence for broader clinical adoption.

## 5 Conclusion

A retrospective analysis was conducted on 26 patients (45 lower limbs) with PDPN who underwent quadruple nerve decompression. Significant efficacy was observed in relieving pain, improving sensory function and increasing nerve conduction velocity. The incidence of post-operative complications was low, indicating that the procedure may be a safe and effective surgical intervention for patients with PDPN.

## Data Availability

The original contributions presented in the study are included in the article/Supplementary Material, further inquiries can be directed to the corresponding author/s.
